# Expanding TREC and KREC Utility in Primary Immunodeficiency Diseases Diagnosis

**DOI:** 10.3389/fimmu.2020.00320

**Published:** 2020-03-03

**Authors:** Ilya Korsunskiy, Oleg Blyuss, Maria Gordukova, Natalia Davydova, Alexey Zaikin, Natalia Zinovieva, Sergey Zimin, Robert Molchanov, Aminat Salpagarova, Alina Eremeeva, Maxim Filipenko, Andrey Prodeus, Anatoliy Korsunskiy, Peter Hsu, Daniel Munblit

**Affiliations:** ^1^Speransky Children's Hospital, Moscow, Russia; ^2^Department of Paediatrics and Paediatric Infectious Diseases, Institute of Child's Health, Sechenov First Moscow State Medical University (Sechenov University), Moscow, Russia; ^3^Wolfson Institute of Preventive Medicine, Queen Mary University of London, London, United Kingdom; ^4^School of Physics, Astronomy and Mathematics, University of Hertfordshire, Hatfield, United Kingdom; ^5^Department of Mathematics and Institute for Women's Health, University College London, London, United Kingdom; ^6^State Institution “Dnipropetrovsk Medical Academy of the Ministry of Health of Ukraine”, Dnipro, Ukraine; ^7^Pharmacogenomic Laboratory, Institute of Chemical Biology and Fundamental Medicine, Novosibirsk, Russia; ^8^Allergy and Immunology, The Kids Research Institute, The Children's Hospital at Westmead, Sydney, NSW, Australia; ^9^The In-vivo Global Network, an Affiliate of the World Universities Network (WUN), New York, NY, United States; ^10^Discipline of Child and Adolescent Health, The University of Sydney, Sydney, NSW, Australia; ^11^Inflammation, Repair and Development Section, Faculty of Medicine, NHLI, Imperial College London, London, United Kingdom; ^12^Solov'ev Research and Clinical Center for Neuropsychiatry, Moscow, Russia

**Keywords:** TREC, KREC, primary immunodeficiency diseases, PID, primary immunodeficiency diseases diagnosis

## Abstract

Primary immunodeficiency diseases (PID) area heterogeneous group of disorders caused by genetic defects of the immune system, which manifest clinically as recurrent infections, autoimmune diseases or malignancies. Early detection of PID remains a challenge, particularly in older children with milder and less specific symptoms. This study aimed to assess TREC and KREC diagnostic ability in PID. Data from children assessed by clinical immunologists at Speransky Children's Hospital, Moscow, Russia with suspected immunodeficiencies were analyzed between May 2013 and August 2016. Peripheral blood samples were sent for TREC/KREC, flow cytometry (CD3, CD4, CD8 and CD19), IgA and IgG analysis. A total of 434 children [189 healthy, 97 with group I and II PID (combined T and B cell immunodeficiencies & well-defined syndromes with immunodeficiency) and 148 group III PID (predominantly antibody deficiencies)] were included. Area under the curve (AUC) for TREC in PID groups I and II diagnosis reached 0.82 (CI = 0.75–0.90), with best model providing sensitivity of 65% and specificity of 92%. Neither TREC, nor KREC had added value in PID group III diagnosis. In this study, the predictive value of TREC and KREC in PID diagnosis was examined. We found that the TREC had some diagnostic utility for groups I and II PID. Possibly, addition of TREC measurements to existing clinical diagnostic algorithms may improve their predictive value. Further investigations on a larger cohort are needed to evaluate TREC/KREC abilities to be used as diagnostic tools on a wider scale.

## Highlights

- ***What is already known about this topic?***
  Assessment of TREC levels is actively used in the screening of severe combined immunodeficiency disorders (SCID).
- ***What does this article add to our knowledge?***
  This study shows that TREC may have a place not just in SCID screening but in the diagnosis of PID.
- ***How does this study impact current management guidelines?***
  Evidence suggests that TREC may be a good addition to already existing diagnostic methods in groups I and II PID diagnosis. It may be particularly useful in less affluent environments with lack of access to flow cytometry.

## Introduction

Primary immunodeficiency diseases (PID) are a heterogeneous group of disorders caused by genetic defects of the immune system, which manifest clinically as recurrent infections, autoimmune diseases or malignancies. The most severe forms of PID, severe combined immune deficiency (SCID) is intensively studied and has been found to be associated with fatal consequences in the first 2 years of life (1, 2).

Most forms of SCID can be detected by measuring the levels of T-cell recombination excision circles (TREC) in dried blood spots using real-time polymerase chain reaction (PCR) (3), while kappa-deleting recombination excision circles (KREC) are used to screen for agammaglobulinemia (4). TREC is a by-product of the T-cell receptor gene recombination, and KREC is a by-product of the B-cell receptor recombination. Low levels of these molecules in T- and B-cells in peripheral blood were shown to be associated with T- and/or B-cell lymphopenia (4). The best possible outcome for patients with SCID can be achieved by timely hematopoietic stem cell transplantation or gene therapy before the development of infectious complications (5, 6), while early diagnosis is associated with a significant increase in treatment effectiveness (5). In patients with agammaglobunemia, the best outcome is achieved by initiating replacement therapy using intravenous or subcutaneous immunoglobulins.

PID influence child health-related quality of life, limiting physical, emotional, social and school functioning (7). Therefore, early detection of not only SCID but all PID patients is vital to improve the chances of appropriate management, in order to significantly reduce potential complications and improve life quality (6, 8).

At present, early detection of PID remains a challenge. This is particularly true in older children and in adults, potentially due to milder and less specific symptoms, a low level of awareness of PID amongst clinicians, as well as unavailability of necessary diagnostic devices such as flow cytometry in hospital laboratories (9, 10). In some countries flow cytometry is not readily available and TREC/KREC analysis may represent a feasible alternative. It is therefore imperative to not only allow diagnosis of patients in low-resource facilities but also develop more cost-effective alternatives. Many studies showed that dried blood spots are robust (11) and useful as a potential alternative sample source for clinical purposes, epidemiological studies, and biobanking (12).

Flow cytometry is a more commonly used but more expensive diagnostic technique for PID detection, when compared with PCR (13). It requires a significant amount of training in highly specialized tertiary centers and therefore cannot be used as a screening tool.

Potential applications of TREC/KREC analysis were highlighted in the reviews by van Zelm and co-authors (14, 15). These included support therapy monitoring, patient classification and newborn screening for PID. Apart from apparent clinical benefits, assessment of B- and T- cell neogenesis in PID patients following stem cell transplantation (16) and KREC assessment in patients presenting with abnormalities in B-cell subsets to explain B-cell compartment aberrancies (17) may improve current state of knowledge.

This pilot study aims to assess diagnostic accuracy of TREC and KREC in children from 0 to 18 years of age with suspected PID.

## Methods

### Study Setting, Eligibility Criteria, and Ethics

In this prospective study, we recruited all children referred by primary care physicians (polyclinic pediatricians) to a tertiary level center (Moscow City Pediatric Hospital #9 named after Speransky, Moscow, Russia) with suspected immunodeficiencies and assessed by board-certified clinical immunologists between May 2013 and August 2016. The investigations and sample collection were conducted following ethical approval by the Speransky Children's Hospital Ethics Committee. Parental written consent was obtained for all participants as part of routine procedure at Speransky Children's Hospital. Parents/guardians were informed of the procedures in lay terms. The study design has been described in detail elsewhere (18).

### Outcome Definition

The primary outcome of interest in this study was PID. We considered that a child has a PID if he or she had PID diagnosed by a physician. The diagnosis of different groups of PID was based on IUIS Phenotypic Classification for Primary Immunodeficiencies (19): group I was defined as immunodeficiencies affecting cellular and humoral immunity; group II corresponded to combined immunodeficiencies with associated or syndromic features, group III was defined as predominantly antibody deficiencies.

### Sample Analysis

Peripheral blood samples were taken by venipuncture during morning hours, aliquoted and sent for complete blood count, flow cytometry, immunoglobulin (IgA, IgG), and TREC/KREC analysis. All blood samples were EDTA-anticoagulated and analyzed on the day of collection to avoid cellular death. Immunoglobulin levels were measured in blood serum.

Sample analysis was performed as described elsewhere (18). In brief, three-four color flow cytometric immunophenotyping with directly labeled monoclonal antibodies was used to determine the following immune cell subsets: CD3-CD19+, CD3-CD(16 + 56)+, CD3+CD4+, and CD3+CD8+ following manufacturer's protocol. Further analysis was performed with a FACS Canto II flow cytometer using FACSDiva v7.0 software (Becton Dickinson). The total leucocyte count and differential was measured with Advia 2120i hematology analyzer (Siemens). The absolute size of each lymphocyte subpopulation was calculated by multiplying the relative size of the lymphocyte subpopulation by the absolute lymphocyte count. Immunoglobulin levels were assessed using a biochemical analyzer Architect C8000 (Abbott, USA, Abbott kits) in accordance with manufacturers' protocol. TREC and KREC assays were performed using real-time PCR with fluorescent hybridization probes and reagents for TREC/KREC assays: T&B PCR kit (ABV-test, Russia) (20), in whole blood. The TREC/KREC levels were assayed in whole blood samples as described previously (16, 18, 20), In brief, DNA was extracted from 100 μl EDTA anticoagulated whole blood by using RIBO-prep nucleic acid extraction kit (Amplisense®, Russia). The Real-time qPCR was performed using CFX 96 Real-Time PCR System (Bio Rad, USA). Amplification of ALB was used to assess correct sampling and quality of DNA extraction, as well as to determine TREC and KREC levels. The number of TREC/KREC copies was calculated per 10^5^ white blood cells, accounting for the quantity of ALB using the formula: [The number of TREC/KREC copies/the number of ALB copies] × 200,000. The normal/cutoff levels of TRECs and KRECs of 1,000 copies/10^5^ cells were used.

### Statistical Analysis

Shapiro-Wilk test was used to assess whether analyzed variables were normally distributed. Since the null hypothesis was not rejected, Spearmen correlation coefficient was used to assess the strength of the correlation between the variables. Sensitivity, specificity and their 95% confidence intervals were computed with stratified bootstrap replicates (21). Area under Receiver Operating Characteristic (ROC)—curve (AUC) calculation was followed by 95% confidence interval as suggested by DeLong et al. (22). The diagnostic accuracy measures used were: sensitivity, specificity, positive predictive value (PPV) and negative predictive value (NPV). TREC/KREC accuracy in diagnosing group I/II and group III PIDs were assessed. The ROC-analysis was performed separately for PID groups I/II and PID group III. The performance characteristics of all lymphocyte subpopulations as well as combinations of TREC and KREC were evaluated and compared in terms of (a) the sensitivity (proportion detected of those with PID) at a fixed specificity (proportion of controls correctly detected not to have PID) and (b) AUC.

Due to TREC levels decreasing with age, every TREC measurement was divided by the corresponding TREC reference interval for the patient's age group prior to analysis.

Results were considered statistically significant if *p*-value was smaller than 0.05. All calculations were done using R package version 3.4.1.

## Results

### Study Population

The data was extracted from clinical notes and the laboratory database of Speransky Children's Hospital. Out of 3,055 patients requiring flow cytometry within the given period of time, due to financial restrictions (those eligible for flow cytometry to be covered by compulsory health insurance in accordance to local regulations. Regulations did not change throughout the study period.), a total of 839 samples were analyzed using flow cytometry and TREC assay and 931 samples were analyzed using flow cytometry and KREC assay. Data on confirmed clinical diagnosis was available from 471 participants. All data points required for TREC/KREC diagnostic properties assessment were available from 434 children and were included in the statistical analysis. Out of 434 children with a doctor's confirmed diagnosis, 189 were immunologically healthy, 97 were group I and II PID patients and 148 were group III PID patients. The following conditions were diagnosed in each subcategory in accordance to International Classification of Diseases, 10th revision (ICD-10) and classified following IUIS Phenotypic Classification for Primary Immunodeficiencies (19): group I (combined immunodeficiencies), group II (immunodeficiency associated with other major defects, ataxia telangiectasia and other specified immunodeficiencies), group III (immunodeficiency with predominantly antibody defects and common variable immunodeficiency). For the sake of the readers' convenience we will use the following terminology in the following sections of the manuscript: “Combined PID” for group I, “Syndromic PID” for group II and “Antibody PID” for group III. The diagnoses were reached using clinical signs and immune phenotype. Genetic testing was not available at the recruitment site.

All the samples were analyzed using flow cytometry, turbidimetry, and TREC/KREC assays and were included into the primary analysis of this study. Demographic data of the participants is presented in Table 1.

**Table 1:** Characteristics of study participants.

**PID Group**	**Total number of patients**	**Clinical diagnosis (number of patients)**	**Age**	**Gender**	
				**Male**	**Female**
Group I (“Combined PID”)Immunodeficiencies affecting cellular and humoral immunity	17	D81 Combined immunodeficiencies (17)	0–12 months 1–6 years 6–12 years 12–18 years	9 2 1 0	5 0 0 0
Group II (“Syndromic PID”)CID with associated or syndromic features	80	D82 Immunodeficiency associated with other major defects (13) D82.1 Di George syndrome (39) D82.4 Hyperimmunoglobulin E syndrome (5) D84.8 Other specified immunodeficiencies (15) G11.3 (8)	0–12 months 1–6 years 6–12 years 12–18 years	7 26 10 4	9 17 6 3
Group III (“Antibody PID”)Predominantly antibody deficiencies	148	D80.0 Immunodeficiency with predominantly antibody defects (4) D80.1 Non-familial hypogammaglobulinaemia (47) D80.2 Selective deficiency of immunoglobulin A (34) D80.3 Selective deficiency of immunoglobulin G (24) D80.4 Selective deficiency of immunoglobulin M (1) D80.5 Immunodeficiency with increased immunoglobulin M (4) D83 Common variable immunodeficiency (34)	0–12 months 1–6 years 6–12 years 12–18 years	6 21 28 38	2 17 21 13
Control group (Healthy children)	226	No clinical diagnosis of PID	0–12 months 1–6 years 6–12 years 12–18 years	9 41 36 25	5 48 33 29

*All codes and diagnoses are in accordance with international classification of diseases, 10th revision (ICD-10)*.

### Descriptive Results of Flow Cytometry and TREC/KREC Testing

**Figure 1:**
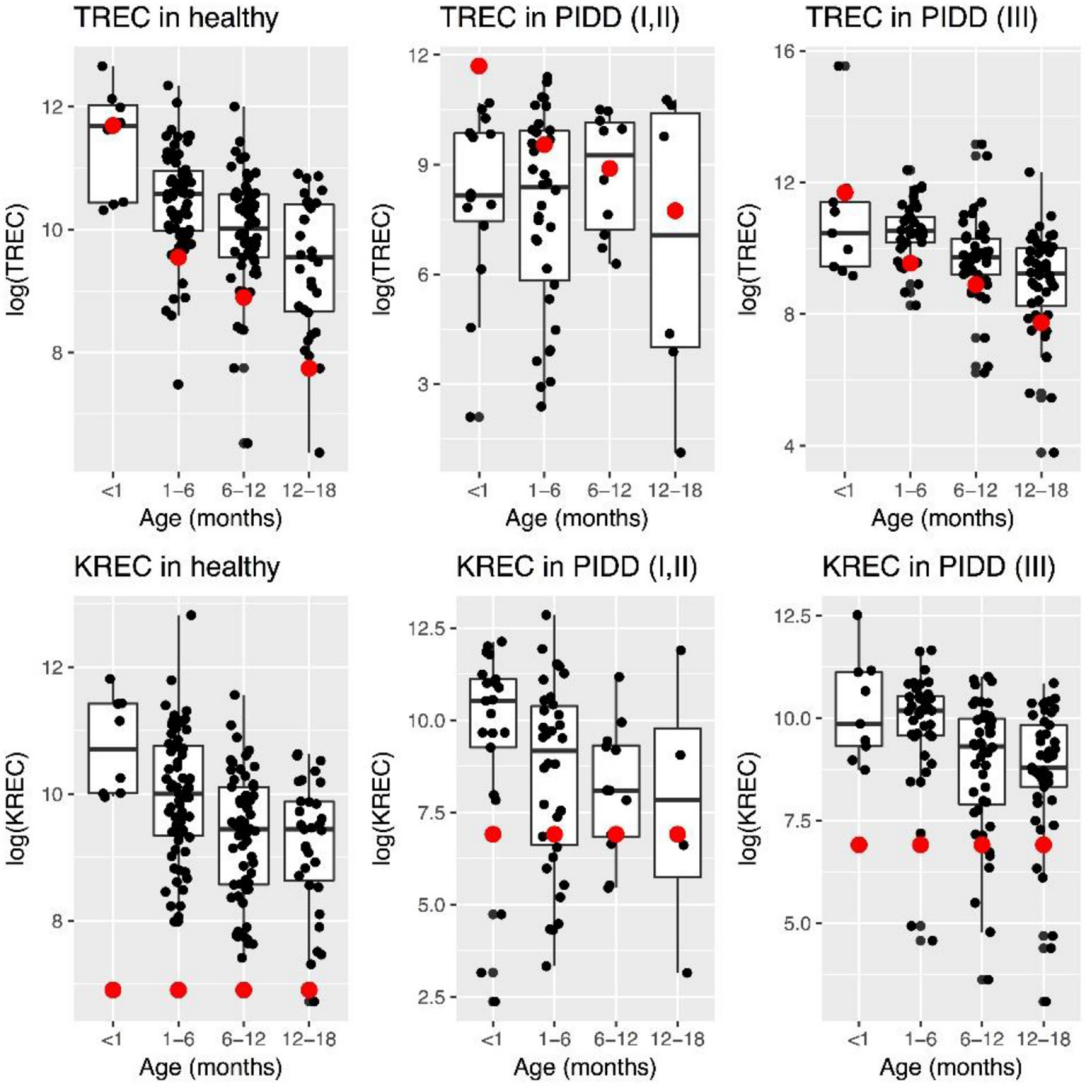
Patterns of change in TREC and KREC log-transformed (natural logarithm) levels in healthy individuals and PID patients at different age. Red dot represents minimal normal level for a given age group.

Levels of TREC decreased with age in healthy children, while this was less evident in “Combined PID” and “Syndromic PID.” Overall TREC levels were lower in “Combined PID” and “Syndromic PID” compared to healthy individuals (Figure 1).

### TREC/KREC Diagnostic Accuracy

**Figure 2:**
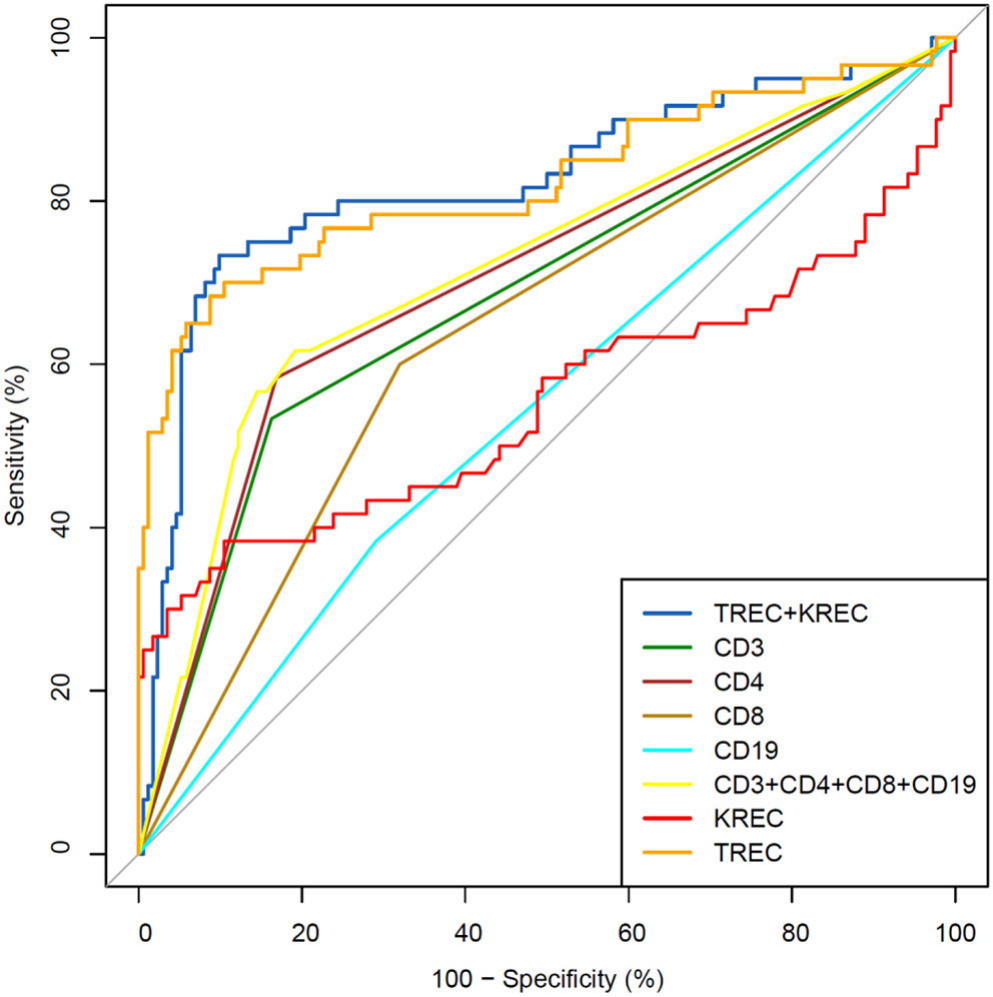
Receiver operating characteristic (ROC) curves for each of lymphocyte subpopulations (CD3, CD4, CD8, and CD19) individually; combined diagnostic ability of all lymphocyte subpopulations and diagnostic ability of TREC and KREC combination in “Combined PID” and “Syndromic PID” diagnosis. Healthy individuals (*n* = 172); children diagnosed with “Combined PID” and “Syndromic PID” (*n* = 60). AUC for TREC and a combination of TREC and KREC = 0.82 (95% CI = 0.75–0.90).

**Figure 3:**
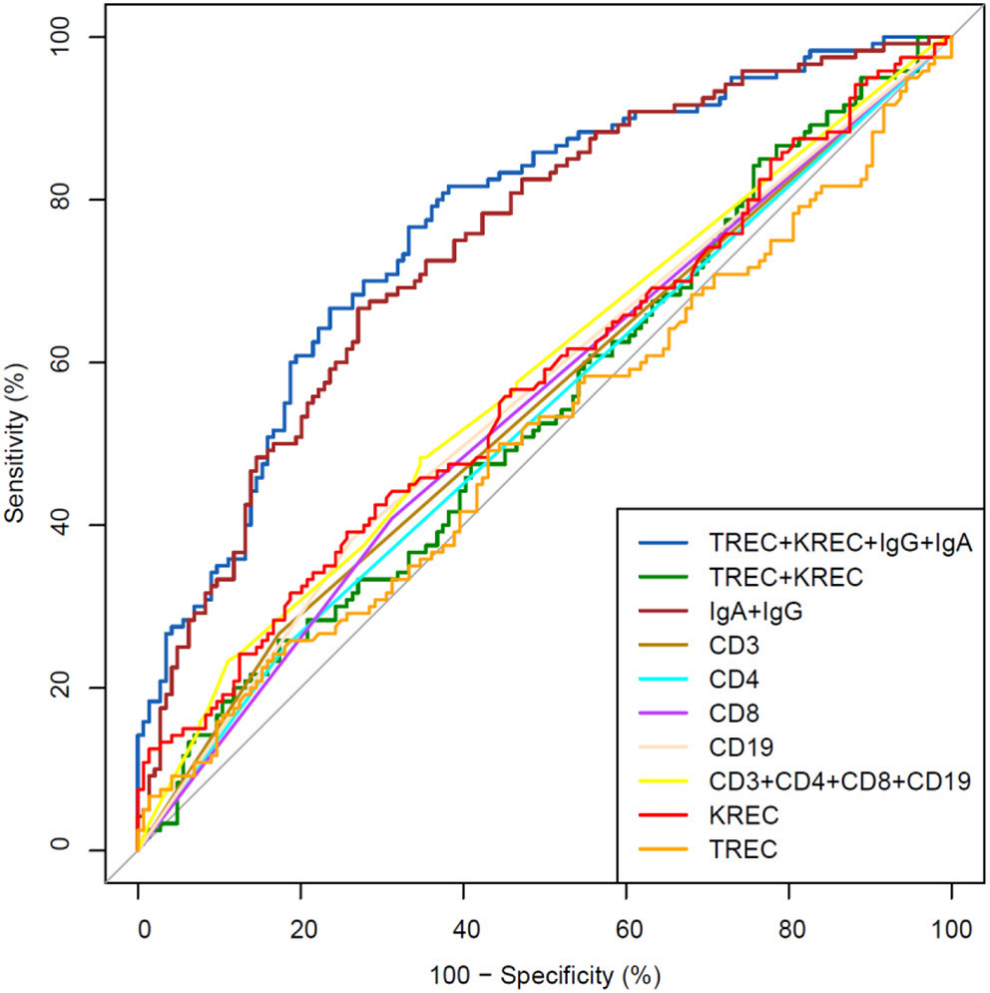
Receiver operating characteristic (ROC) curves for each of lymphocyte subpopulations (CD3, CD4, CD8, and CD19) individually; combined diagnostic ability of all lymphocyte subpopulations; IgA and IgG combined; TREC and KREC combined and diagnostic ability of TREC, KREC, IgA, and IgG combination in “Antibody PID” diagnosis. Healthy individuals (*n* = 144); children diagnosed with “Antibody PID” (*n* = 120). IgA, IgG, TREC, and KREC AUC = 0.77 (95% CI = 0.71–0.82).

The area under the curve for lymphocyte subpopulations (CD 3, 4, 8, and 19), immunoglobulins (IgA, IgG) and TREC/KREC levels were assessed. Separate analyses were undertaken for “Combined PID” and “Syndromic PID” (Figure 2) and “Antibody PID” (Figure 3). The same analysis was performed to assess ability of TREC/KREC to differentiate between “Combined PID,” “Syndromic PID,” and “Antibody PID.”

AUC for TREC and a combination of TREC and KREC for “Combined PID” and “Syndromic PID” diagnosis was 0.82 (95% CI = 0.75–0.90). As KREC did not add value to the model's predictive capacity, the data for TREC is presented (Table 2). The cutoff point of a probability of 0.4 showed the best diagnostic accuracy with regards to the sensitivity and specificity (65 and 92%), *J* = 57.4.

**Table 2:** Diagnostic accuracy measures for different cutoff points of the predicted probabilities for TREC in “Combined PID” and “Syndromic PID” diagnosis.

**Cutoff point (probability)**	**PPV (%)**	**NPV (%)**	**Sensitivity (%)**	**Specificity (%)**	**Youden index**
0.05	28	92	97	13	9.5
0.1	29	90	93	21	14.2
0.15	32	90	90	32	22
0.2	33	89	85	41	25.7
0.25	37	88	78	54	31.8
0.3	46	90	78	67	45.7
0.35	59	89	72	83	54.3
**0.4**	**75**	**88**	**65**	**92**	**57.4**

*Optimum cut-off point based on maximum value of the J index is presented in bold*.

A combination of IgA, IgG, TREC and KREC demonstrated the best AUC 0.77 (95% CI = 0.71–0.82) for “Antibody PID” diagnosis but neither TREC or KREC nor combination of two yielded good diagnostic ability (AUC 0.54, 95% CI = 0.47–0.61), Table 3. TREC and KREC combination did not demonstrate any added value to immunoglobulin level detection.

**Table 3:** Diagnostic accuracy measures for different cutoff points of the predicted probabilities for a combination of TREC and KREC in “Antibody PID” diagnosis.

**Cutoff point (probability)**	**PPV (%)**	**NPV (%)**	**Sensitivity (%)**	**Specificity (%)**	**Youden index**
0.35	46	75	98	4	2.5
0.4	46	64	93	11	3.6
0.45	47	56	61	42	2.5
**0.5**	**57**	**56**	**14**	**91**	**5.2**
0.55	42	54	4	95	0
0.6	50	55	2	99	0.3

*Optimum cut-off point based on maximum value of the J index is presented in bold*.

**Figure 4:**
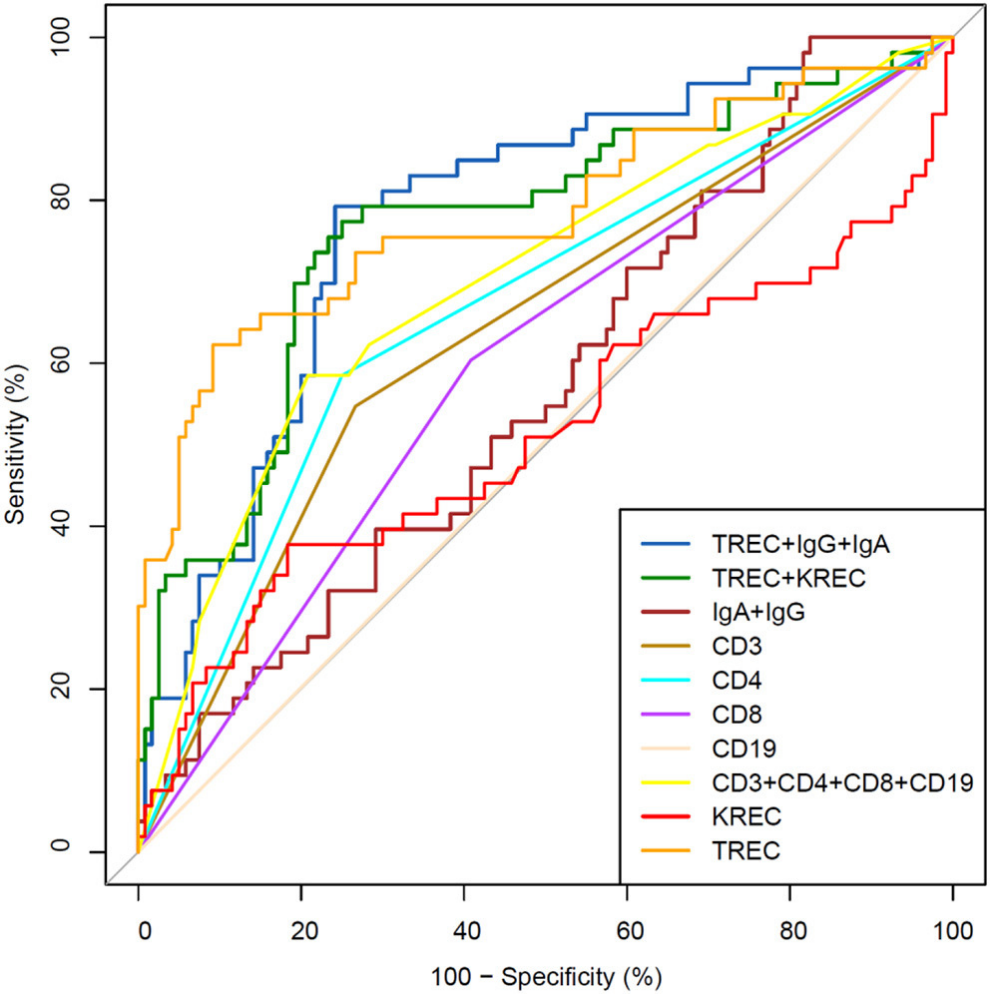
Receiver operating characteristic (ROC) curves for each of lymphocyte subpopulations (CD3, CD4, CD8, and CD19) individually; combined diagnostic ability of all lymphocyte subpopulations; IgA and IgG combined; TREC and KREC combined and diagnostic ability of TREC, KREC, IgA, and IgG combination in differentiating between “Combined PID” and “Syndromic PID” and “Antibody PID.” “Combined PID” and “Syndromic PID” individuals (*n* = 53); children diagnosed with “Antibody PID” (*n* = 120). TREC AUC = 0.79 (95% CI = 0.70–0.87).

We then performed analysis to study ability of TREC/KREC to differentiate between “Combined PID,” “Syndromic PID,” and “Antibody PID.” As KREC did not add value to the predictive model, the predictive model based on TREC levels is presented (Figure 4). The AUC for TREC in “Combined PID” and “Syndromic PID” diagnosis was 0.79 (95% CI = 0.70–0.87). A cutoff point of a probability of 0.4 showed the best diagnostic accuracy with regards to the sensitivity and specificity (66 and 84%; (Table 4), *J* = 50.2.

**Table 4:** Differential diagnosis for “combined PID” and “syndromic PID” and “antibody PID.” Accuracy measures for different cutoff points of the predicted probabilities for TREC.

**Cutoff point (probability)**	**PPV (%)**	**NPV (%)**	**Sensitivity (%)**	**Specificity (%)**	**Youden index**
0.05	31	80	96	7	2.9
0.1	33	89	96	13	9.5
0.15	34	88	94	18	12.6
0.2	36	89	93	28	20
0.25	38	88	89	35	23.7
0.3	40	84	79	47	25.9
0.35	51	86	76	68	43.8
**0.4**	**65**	**85**	**66**	**84**	**50.2**

*Optimum cut-off point based on maximum value of the J index is presented in bold*.

## Discussion

In this study, we tested the accuracy of TREC and KREC in PID diagnosis. The models showed decent utility of TREC in the diagnosis of “Combined PID” and “Syndromic PID.” To our knowledge, this is the first attempt to use TREC/KREC not in SCID screening but in PID diagnostics.

PID is a large group of disorders encompassing more than 400 conditions affecting development and/or functioning of the immune system (23). Flow cytometry is a sensitive and important tool in evaluating the immune system function and in PID diagnosis (24). However, it is expensive, not easily available in developing countries and requires appropriate training and equipment. TREC and KREC may represent cheaper alternatives and/or add value to PID diagnosis and screening. Low cost methodology can be used in small laboratories and rural settings, where complex and expensive tools are unavailable, to provide access to primary PID evaluation.

Prior studies have noted the need in screening tool for early SCID diagnosis, to reduce the risk of infections and organ damage (6, 25). Early diagnosis is particularly important as lack of early treatment is associated with severe complications and increased mortality rates (26). TREC is a common screening approach used for early SCID detection around the globe, providing a good combination of sensitivity and specificity (27, 28) with high cost-effectiveness (29). KREC's role in early screening is still debatable but some data suggest that it may add value in certain cases (30). While TREC's indispensability in SCID screening is obvious, very little can be found in the literature on the question of TREC/KREC use in other PID diagnosis or their use as a screening tool (31–33). Previous attempts to this end have predominantly focused on Common Variable Immunodeficiency (CVID).

We hypothesized, that TREC and KREC may be adopted as a surrogate method of PID diagnosis. Our models demonstrated good AUCs indicating the potential of TREC to be used as an additional tool in PID diagnosis. Our data show that when TREC is used for differentiation between “Combined PID” and “Syndromic PID” patients and healthy individuals, the cut-off point probability of 0.4 provides high specificity (92%) with acceptable sensitivity (65%), further supporting our hypothesis that use of TREC may have its place in PID diagnosis. TREC may serve as an addition to existing tests and may be used as a prerequisite to flow cytometry. We did not use any clinical questionnaires in this study but expect that laboratory findings combined with additional clinical data using standardized instruments may facilitate development of a stronger diagnostic model, improving utility.

TREC and KREC whether individually or combined, did not show good diagnostic ability in diagnosing “Antibody PID.” However, this result was expected, with immunoglobulin measurement having the primary role in aiding the diagnosis. Our further analysis highlights potential for TREC level assessment in discrimination between “Combined PID,” “Syndromic PID,” and “Antibody PID.” When a cut-off of 0.4-point probability was used, TREC reached specificity of 84%. At present, the wide variety and lack of specificity of PID clinical manifestations, do not allow physicians to determine the exact area of immune defect during initial clinical examination. Thus, the extensiveness of the primary laboratory examination in a patient with suspected PID is often determined by subjective clinical criteria, based on physician expertise. Laboratory tests normally used vary from immunoglobulin level assessment to assessment of a wide range of lymphocyte subpopulations. There are no predefined universal guidelines on how detailed the laboratory analysis should be, while selection of diagnostic tests depends on the given clinical settings and clinical immunologist. Normally, if initial tests reveal deviations in humoral immunity the next logical step is cellular immunity assessment to exclude combined immunodeficiency and diagnose “Antibody PID.” Our data suggests that TREC appears to be a useful additional tool to aid in the differentiation between combined immunodeficiency and antibody deficiencies, particularly in the settings with limited access to flow cytometry. The qualitative method of TREC/KREC assessment is easy and can be applied equally well to both whole blood and Guthrie cards, as DNA extraction can be performed on either of these samples.

A few diagnostic algorithms/approaches to children with suspected PID were proposed. Among them excellent algorithm from Dutch immunologist Esther DeVries', based on a pattern recognition approach and decision trees (34), and the Jeffrey Modell Foundation's 4 steps (35). We do not propose changes to existing clinical approaches in routine PID diagnosis and fully acknowledge that TREC/KREC should not be used as a replacement of flow cytometry. Addition of TREC/KREC measurements to aforementioned clinical approaches, nevertheless, may improve predictive capacity of the tools, which is worth further investigation.

The main limitation of this study is related to the use of ICD-10 classification for PID diagnosis. The same ICD-10 code may sometimes include heterogenous group of immunodeficiencies. Genetic testing would be a preferable option; however, this was not available for most of the patients due to economical restrictions. Another limitation is the lack of Guthrie card use in our study with all samples analyzed using whole blood, which does not allow for a result extrapolation. No difference is expected, however, between DNA extraction from the whole blood sample and dried blood spot. Recruitment of patients of three PID groups only, may also be considered a limitation, even though it is unlikely to have influenced the outcomes of this study. We acknowledge that participants defined as “immunologically healthy” in this study can be considered as “healthy” to a high degree of certainty, but no information on naive cells and memory cells were collected. It is clear, however, that “immunologically healthy” participants in this study do not belong to Group I (“Combined PID”). Our study would also have benefited from assessment of TREC/KREC diagnostic accuracy in patients with a particular subtype of PID [e.g., X-linked agammaglobulinemia (XLA)], but it was not possible due to restricted number of patients. This should be addressed in future research.

In conclusion, we found evidence that TREC may have a place in aiding PID diagnosis. The models showed decent diagnostic accuracy measures for TREC in diagnosing “Combined PID” and “Syndromic PID”. Further investigations in a larger cohort in combination with addition of genetic diagnoses and/or questionnaires focused on clinical symptoms are needed to improve diagnostic performance and to further evaluate TREC potential on a wider scale. It is too premature to draw definitive conclusions, but with a few diagnostic algorithms available (e.g., Esther DeVries' and Jeffrey Modell Foundation's 4 steps), addition of TREC to such algorithms may allow for an improved predictive ability. We would like to stress that TREC-based PID diagnosis may be particularly important in the recourse-limited countries and further research will benefit children in these settings.

## Data Availability Statement

The datasets generated for this study are available on request to the corresponding author.

## Ethics Statement

The studies involving human participants were reviewed and approved by Speransky Children's Hospital Ethics Committee. Written informed consent to participate in this study was provided by the participants' legal guardian/next of kin.

## Author Contributions

IK, MF, AP, AK, and DM conceived and designed the experiments and study analysis. MG and ND performed the experiments. IK, NZ, SZ, and AS collected, extracted, and sorted the data. OB, RM, and AZ analyzed the data. AE reviewed additional available evidence on the matter. IK, PH, and DM wrote the manuscript.

### Conflict of Interest

MG, MF, and IK are board members for ABV-test. MG, MF, AP, and IK has a patent with ABV-test. DM has given paid lectures for Merck Sharp & Dohme (MSD) and Bayer. DM also is a member of ILSI Europe: Immune Competence Across Lifespan: Impact of Nutrition on Immune Competence and its Consequences Later in Life expert group. The remaining authors declare that the research was conducted in the absence of any commercial or financial relationships that could be construed as a potential conflict of interest.

## Abbreviations

AUC: Area under receiver operating characteristic curve
CD: cluster of differentiation
CVID: Common Variable Immunodeficiency
DNA: Deoxyribonucleic acid
ICD-10: International Classification of Diseases, 10th Revision
Ig: immunoglobulin
IUIS: International Union of Immunological Societies
KREC: kappa-deleting element recombination circle
PCR: polymerase chain reaction
PID: Primary immunodeficiency diseases
ROC: Receiver Operating Curve
SCID: Severe Combined Immune Deficiency
TREC: T-cell recombination excision circles.
